# Proactive strategies to optimize engagement of Black, Hispanic/Latinx, transgender, and nonbinary individuals in a trial of a novel agent for HIV pre-exposure prophylaxis (PrEP)

**DOI:** 10.1371/journal.pone.0267780

**Published:** 2022-06-03

**Authors:** Michelle Cespedes, Moupali Das, J. Carlo Hojilla, Jill Blumenthal, Karam Mounzer, Moti Ramgopal, Theo Hodge, Thiago S. Torres, Charles Peterson, Senzokuhle Shibase, Ayana Elliott, A. C. Demidont, Larkin Callaghan, C. Chauncey Watson, Christoph Carter, Alex Kintu, Jared M. Baeten, Onyema Ogbuagu

**Affiliations:** 1 Division of Infectious Disease, Icahn School of Medicine at Mount Sinai, New York, NY, United States of America; 2 Gilead Sciences, Foster City, CA, United States of America; 3 Department of Medicine, University of California San Diego, San Diego, CA, United States of America; 4 Philadelphia FIGHT Community Health Centers, Philadelphia, PA, United States of America; 5 Midway Research Center, Fort Pierce, FL, United States of America; 6 Washington Health Institute, Washington, DC, United States of America; 7 Instituto Nacional de Infectologia Evandro Chagas, Fundação Oswaldo Cruz, Rio de Janeiro, Brazil; 8 Department of Medicine, College of Medicine, University of Illinois Chicago, Chicago, IL, United States of America; 9 National LGBTI Health Campaign, Johannesburg, South Africa; 10 Department of Internal Medicine, Section of Infectious Diseases, Yale School of Medicine, New Haven, CT, United States of America; University of Toronto, CANADA

## Abstract

**Introduction:**

Black and Hispanic/Latinx cisgender men who have sex with men (MSM), transgender women, transgender men, and gender nonbinary (TGNB) individuals have been historically underrepresented in HIV pre-exposure prophylaxis (PrEP) clinical trials. There is an urgent need for ongoing engagement with communities that have been the most impacted by HIV and diverse representation in clinical trials. Here we describe strategic approaches undertaken in the PURPOSE 2 trial to optimize engagement of underrepresented individuals.

**Methods and results:**

PURPOSE 2 is an ongoing Phase 3 trial evaluating the safety and efficacy of lenacapavir as PrEP in cisgender MSM and TGNB individuals. In PURPOSE 2, we used a multipronged approach aimed at enriching participation of underrepresented individuals. We conducted a review to identify evidence-informed recommendations from literature, engaged with stakeholders, and established the Global Community Advisory and Accountability Group (GCAG) to represent the needs of the community. Insights from stakeholders and GCAG members resulted in an expansion of the study population to include transgender men, gender nonbinary persons, and adolescents, and evaluation of population-specific outcomes. Feedback from stakeholders and GCAG members also informed investigator and site selection; these were selected based on prior experience working with persons from diverse racial, ethnic and gender identities, and estimates of local HIV incidence. Site selection was also expanded to include community-based clinics with services tailored towards Black, Hispanic/Latinx, and TGNB populations. We established a study-wide recruitment goal of 50% Black MSM and 20% Hispanic/Latinx MSM in US sites and 20% transgender women globally. Site-specific recruitment goals were also developed based on local demographics and HIV incidence. Mandatory trainings included Good Participatory Practice guidelines, gender inclusivity, and antiracism.

**Conclusion:**

While further work is needed to achieve equitable representation, the strategies we describe may serve as a framework for future clinical trials.

**Trial registration:**

Clinical Trial Number: NCT04925752.

## Introduction

Historically underrepresented individuals, particularly Black and Hispanic/Latinx cisgender gay, bisexual, and other men who have sex with men (MSM) and transgender women, transgender men, and gender nonbinary (TGNB) individuals, have a disproportionate burden of HIV [1, 2]. In the US, HIV incidence among Black and Hispanic/Latinx MSM is estimated to be eight and four times higher than their White counterparts, respectively [3]. In 2019, over half of all new HIV diagnoses were in Southern states, of which 47% were among Black MSM and 28% among Hispanic/Latinx MSM [4]. Estimates suggest HIV prevalence among transgender women is as high as 40% in some US cities, with Black and Hispanic/Latinx transgender women bearing the greatest burden [5]. Globally, transgender women have 49-fold greater odds of having HIV compared with cisgender individuals [6]. Less is known about the HIV burden in transgender men, but available data from US cohorts suggest an HIV prevalence of up to 11% among transgender MSM [7, 8].

Adolescents, particularly those in racial, ethnic, gender, and sexual minority groups, similarly experience high rates of HIV infection and face additional challenges to accessing healthcare services [9]. An estimated 1.75 million adolescents are living with HIV globally [10], and among US adolescents diagnosed with HIV, 5% were in persons aged 15–17 years [4]. Despite significant disparities in ongoing HIV incidence, uptake of pre-exposure prophylaxis (PrEP) in these key populations have been limited. This is noteworthy as PrEP is highly effective in reducing the risk of acquiring HIV [11], but estimates suggest that only approximately 20% of the 1.2 million estimated people who would benefit from PrEP in the US are currently using it [12]. Globally, the proportion of persons on PrEP is even lower [13, 14] and among individuals who initiate PrEP, many discontinue within a few months of starting the regimen [15, 16].

The factors driving inequities in PrEP uptake and persistence in key populations are complex and multifactorial. However, underrepresentation of these communities in clinical research, as well as stigma, discrimination, and transphobia in research and medical settings have perpetuated mistrust [17–19]. Engagement with communities that have the highest HIV burden, as well as advocates, providers, and investigators who represent these communities, is critical, [20, 21] and there is renewed focus on designing clinical trials that have diverse representation. In this paper, we provide a detailed description of the strategic evidence-informed approaches aimed at optimizing engagement of historically underrepresented individuals undertaken ahead of the initiation of a recently launched Phase 3 trial of a novel PrEP agent.

## Methods

PURPOSE 2 (GS-US-528-9023; NCT04925752) is an ongoing Phase 3 clinical trial evaluating the safety and efficacy of lenacapavir (LEN) as PrEP for preventing HIV-1 infection in cisgender MSM and TGNB individuals sponsored by Gilead Sciences. A separate study, PURPOSE 1 (GS-US-412-5624; NCT04994509), is evaluating LEN in cisgender women. LEN is a first-in-class capsid inhibitor, which disrupts HIV capsid and viral replication in multiple steps including nuclear entry and capsid disassembly prior to HIV integration and is administered subcutaneously every six months [22]. In PURPOSE 2, participants are randomized in a 2:1 ratio to receive subcutaneous LEN every 26 weeks plus daily oral placebo or daily oral emtricitabine/tenofovir disoproxil fumarate plus a placebo subcutaneous injection every 26 weeks. The trial started in June 2021 with study sites in Brazil, Peru, South Africa, and the US, and will enroll approximately 3,000 individuals.

In planning for PURPOSE 2, the study team (defined as the sponsor, Gilead Sciences, and global collaborators) used a multipronged approach aimed at intentionally enriching participation of historically underrepresented individuals. We conducted a review of publications to identify effective strategies to increase enrollment of Black and Hispanic/Latinx MSM and TGNB individuals, particularly in HIV treatment and prevention studies. We also engaged with community and patient stakeholders in the US and globally through several community forums, roundtable discussions, and individual meetings to understand community preferences, concerns, and challenges prior to protocol development. Input from these meetings were summarized and incorporated along with recommendations from the literature. The Global Community Advisory and Accountability Group (GCAG) was subsequently established to provide ongoing input on the needs of the community, accountability, and to serve as a resource to the study team and site investigators and staff. In collaboration with members of the GCAG, we developed criteria for site selection and demographic inclusion goals. We also established mandatory trainings for all individuals involved in the study that encompassed Good Participatory Practice (GPP) guidelines, which provides a framework for building effective partnerships with key stakeholders in research [23]; gender inclusivity; and antiracism. The PURPOSE 2 study protocol was approved by institutional review boards/ethics committees (IRB/EC) at each site and by relevant regulatory agencies in each country where recruitment has started, and IRB/EC and regulatory review are continuing in pending countries and sites. All participants provide written informed consent prior to enrollment and when adolescent enrollment initiates, adolescents will provide assent and/or parental consent, as appropriate.

## Results

### Evidence-informed recommendations from literature

The study team reviewed publications on approaches to address disparities in enrollment and retention in clinical trials, including global consensus documents related to the conduct of HIV prevention clinical trials [23, 24]. Key elements to inform new trials included intentional engagement with community stakeholders and advocates, including the establishment of a trial-specific community advisory and accountability group [25–29]. Recommendations also underscored judicious site selection by prioritizing study sites with culturally-sensitive investigators and staff, with prioritization for sites that have local community advisory boards and strong relationships with community-based organizations focused on HIV prevention in key populations [30–32]. Of particular importance, site investigators and/or staff should include individuals from the communities represented in the study [30, 32–34]. Lastly, findings from our review also emphasized the importance of culturally-relevant study design and implementation, including gender affirming practices [18, 35]. These strategies include TGNB-inclusive recruitment materials; gender identity screening and organ inventory; inclusive, trauma-informed sexual history taking guidelines; assessment of intimate partner violence and substance use, and referral to appropriate support services; and more accurately describing TGNB participant inclusion and exclusion criteria. These recommendations from the literature were incorporated into the design and implementation of the PURPOSE 2 study.

### Stakeholder engagement and establishing the GCAG

**Fig 1:**
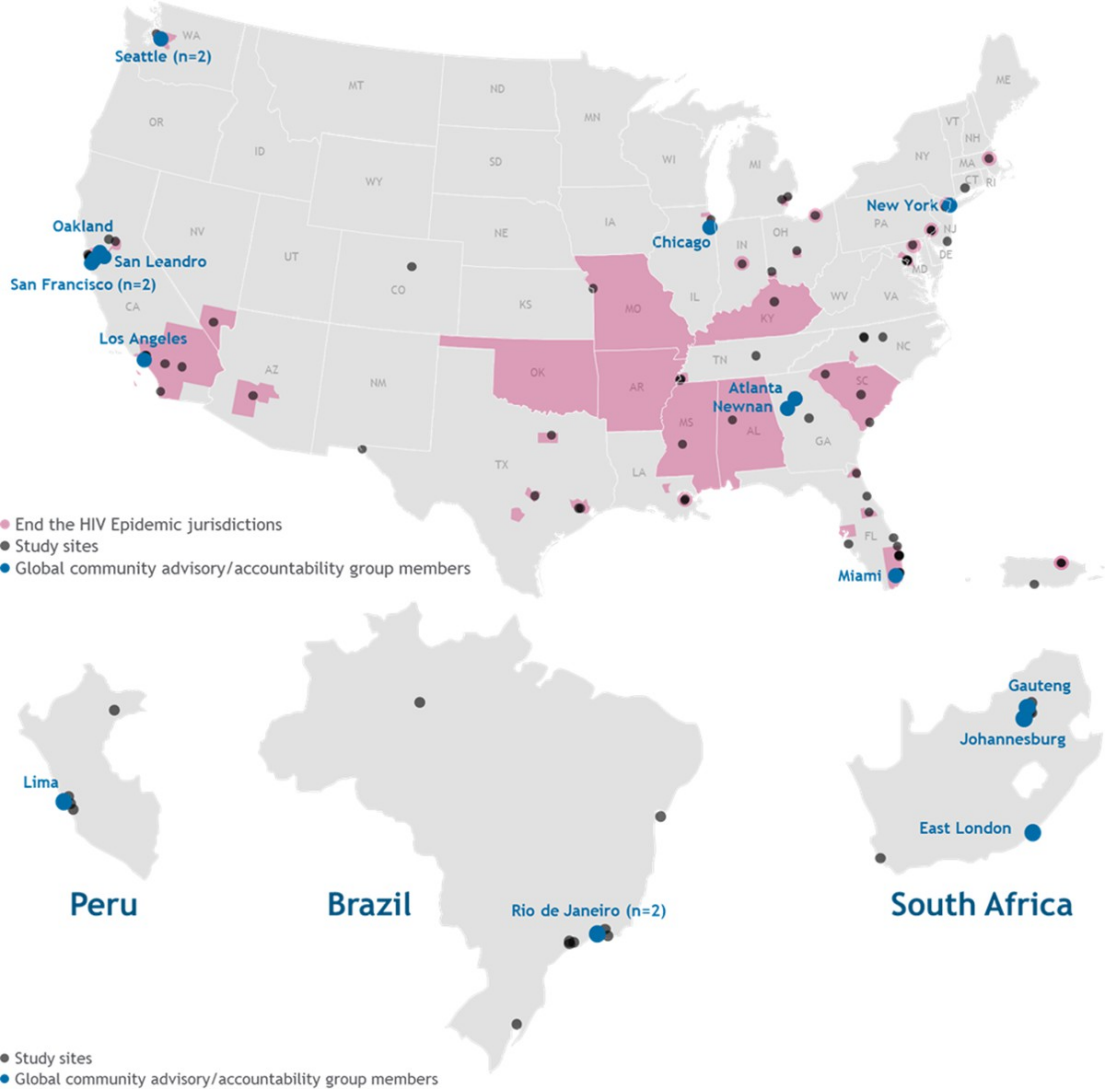
PURPOSE 2 clinical trial study sites and locations of Global Community Advisory and Accountability Group (GCAG) members.

We utilized a two-pronged approach to engage with stakeholders. First, the study team reached out to global advocates, scientists, community representatives, policymakers, and others to request initial feedback on the trial design. We also worked with these initial broader stakeholders to identify and form relationships with advocates who had clinical trial expertise and were from the communities we wished to represent in the study. Building on these relationships, we then established the GCAG to provide ongoing community input on the study. Patient and community advocates who wished to participate in the GCAG were asked to complete an online application. Members were selected based on their personal lived experience as well as HIV research and clinical trial advisory expertise, experience in community engagement with the priority demographic populations for the study, and from recommendations from key community leaders, patient advocates, and site investigators. Prior studies that examined the implementation community engagement across various HIV prevention trials emphasized early involvement and broad representation of community stakeholders as critical to building trust and meaningful collaboration [20, 21]. Thus, GCAG members were involved in each step of the study, including protocol development, site selection, development of participant-facing materials, and study implementation. Additionally, we ensured that the individuals selected to join the GCAG reflected the priority recruitment race, ethnic, and gender demographic goals of the study as well as the geography of the trial sites (Fig 1). The group is compensated for their time and is currently comprised of 18 members.

Insights from meetings with broader stakeholders and the GCAG resulted in meaningful changes to the study (Table 1). For example, the trial’s population was expanded from MSM and transgender women to include transgender men and gender nonbinary individuals who have sex with men. GCAG members made recommendations regarding site and investigator selection, and the inclusion of adolescents in the clinical trial. GCAG members also highlighted concerns that TGNB individuals may have regarding drug-drug interactions between gender-affirming hormone therapy (GAHT) and drugs for HIV prevention, and strongly recommended conducting evaluations of exposure in persons on GAHT within the trial. Moreover, stakeholders recommended specific language and descriptions to improve our inclusion criteria, conduct an organ inventory to assess pregnancy potential, and revise our sexual behavior questionnaires to better reflect the practices of our TGNB participants. Stakeholders also recommended allowing those participants who were assigned female sex at birth and had reproductive potential to remain in the study if they became pregnant, consistent with the evolving consensus on the inclusion of pregnant people in PrEP trials [36] and our plans for the accompanying PURPOSE 1 study.

**Table 1:** Changes to the PURPOSE 2 study design and protocol based on stakeholder and Global Community Advisory and Accountability (GCAG) feedback.

Stakeholder and GCAG Input	Changes Made
Transgender men and gender nonbinary people have been excluded from HIV prevention research	• Inclusion of transgender men and gender nonbinary Individuals • Selection of research sites with experience in engaging transgender men in prevention research
Proactive efforts are needed to ensure inclusion of Black MSM	• Implementation of site-specific recruitment plans designed to facilitate a goal of enrolling 50% Black MSM in the US
Inclusion of diverse study staff is important and often neglected	• Outreach to and selection of investigators and study sites with staff who are representative of the study’s priority participant populations
Many adolescents, especially transgender adolescents, are particularly vulnerable and in need of HIV prevention options	• Inclusion of adolescents ages 16–17 in the study protocol
Participants assigned female sex at birth should not be removed from the study if they become pregnant	• Modification of the protocol to allow participants who become pregnant to remain on study drug after re-consent
Participants who acquire HIV need active connection to HIV care	• Protocol written to facilitate rapid ART start • Continued follow-up in the study until virologic suppression is achieved
Gender-affirming surgical history must be collected in a manner that is accurate and affirming	• Addition of an organ inventory to medical history
Information on gender and sexual behaviors must be obtained in a way that is inclusive and affirming	*De novo* development of sexual behavior questionnaires with skip logic based on gender identity and organ inventory
Concerns about interactions between PrEP and GAHT continue to be a barrier to PrEP for some transgender people	• Discussion about lack of interactions expected between PrEP drugs and GAHT in the protocol and consent • Rigorous collection of concomitant GAHT • Inclusion of LEN and GAHT drug-drug interaction analyses

MSM = men who have sex with men; ART = antiretroviral therapy; PrEP = pre-exposure prophylaxis; GAHT = gender-affirming hormone therapy; LEN = lenacapavir

### Site selection

The PURPOSE 2 trial uses a novel counterfactual study design. In this design, efficacy is estimated by comparing HIV incidence among participants on the study drug with the background HIV incidence, which is calculated using a recent infection testing algorithm from persons who test positive on a recency assay for HIV in the cohort screened for randomization into the study [37, 38]. This design necessitates identifying sites in locations with high rates of new HIV infection. However, input from stakeholders and the GCAG emphasized the importance of identifying investigators within those settings who have expertise in engaging with individuals from our key populations. Thus, there were two main factors that the study team considered in the selection of study sites: 1) their prior work with individuals from diverse racial, ethnic and gender backgrounds and 2) local estimates of HIV incidence.

Site selection began with a standard feasibility survey that was enhanced to include questions about the site’s staff demographics; cultural sensitivity; and expertise in working with racial, ethnic, and gender-diverse groups. Sites were asked to name the community-based organizations and groups they would work with to support the recruitment of these key populations. Sites were also asked about their prior work with community advisors and advocates, and whether they had their own standing community advisory groups. Site selection consideration was expanded beyond HIV clinical treatment trial sites to include community-based clinical research sites; Federally Qualified Health Centers; and other community-based clinics with services tailored to Black, Hispanic/Latinx, and TGNB populations. We focused on increasing the number of sites that provided gender-affirming care, as well as sites and investigators with expertise in HIV prevention for adolescent populations.

We also considered geographic regions that were the most disproportionately affected by HIV so we could engage with persons who could benefit from PrEP. Site selection in the US aimed to reflect the high-priority municipalities and regions identified in the Ending the HIV Epidemic Initiative, where more than 50% of new HIV infections occurred in 2016–2017 (Fig 1) [39, 40]. Selection of sites in the southern US was prioritized, given the high burden of HIV in the region, and potential sites outside the US were identified using historical HIV incidence data.

### Study-wide demographic recruitment goals and site-specific recruitment plans

The study team identified study-wide demographic recruitment goals to support robust representation of key populations. In consultation with stakeholders and GCAG members, we set a goal of enrolling 50% Black MSM and 20% Hispanic/Latinx MSM across US study sites. These benchmarks were informed by recommendations from the HPTN Black Caucus Scientific Report [41] and HIV incidence surveillance data in Hispanic/Latinx MSM [4]. Globally, we set a goal of enrolling 20% transgender women, based on HIV prevalence estimates among transgender women in the countries included in the PURPOSE 2 trial [42–44]. We did not specify goals for transgender men or gender nonbinary individuals due to a lack of granular data regarding HIV incidence in these populations at the local level.

To facilitate recruitment of diverse study participants at each site and achieve our study-wide recruitment goals, we also developed site-specific recruitment plans in collaboration with each site investigator and their research team. Sites have individualized recruitment goals that reflect the demographics of each locale and the HIV incidence in MSM in the local jurisdiction. Sites with declining HIV incidence in MSM (i.e., <1.5 per 100 person-years) will focus on the recruitment of TGNB individuals and those with expertise in recruiting adolescents are encouraged to focus on this age group; however, no specific age goals were set.

During the screening and enrollment phase of the trial, the study team will review the overall metrics for each demographic goal weekly. If needed, the site-specific recruitment plans and the order of site activation (regionally and globally) will be adjusted to achieve overall recruitment goals.

### Training modules on GPP, gender inclusivity, and antiracism

The PURPOSE 2 study team wanted to ensure that all internal team members, external site investigators and staff, and the contract research organization and other vendors were trained on cross-cultural humility and were competent on key issues. We developed three trainings modules focused on 1) GPP; 2) cultural humility and competence in research with TGNB individuals; and 3) racism and implicit bias in clinical trials. These modules were delivered live to internal study teams and recorded and distributed broadly to all investigators, staff, and vendors. The modules were also uploaded to the study’s online portal for on-demand viewing. Completion of trainings is mandatory in the onboarding of new internal and external research staff.

### Clinical trial website and online approaches

Stakeholders provided feedback on the importance of a centralized online location for participants, site investigators and staff, GCAG members, medical professionals, and the general public to access clinical trial information. The study team created a website (http://www.purposestudies.com) to address these needs and house information about the PURPOSE clinical trials. The website includes a site locator, allowing individuals with interest in participating to enter their ZIP code or location to find clinical trial sites in their area. The website will also host study updates, data presentations at scientific conferences, regulatory updates and communications, media coverage, and other study-related information. The study team developed the website to increase awareness, promote recruitment, support continued engagement, and provide updated information for all those interested in HIV prevention clinical trials.

## Discussion

Equitable representation of Black and Hispanic/Latinx MSM and TGNB individuals in HIV PrEP clinical trials is imperative. These populations are the most disproportionately affected by HIV but have been historically underrepresented in clinical trials. Regulatory and funding agencies have implemented various national-level policies to improve participation of marginalized communities in research [45, 46], but these efforts have not resulted in meaningful changes [47–50]. To address the limitations of prior PrEP trials, the PURPOSE 2 study team used novel and intentional approaches to plan for engagement of these key populations.

Historical abuses and unethical treatment of minorities continue to influence people’s perceptions of research [20, 51, 52]. Individuals have described apprehensions around being experimented on and others have expressed skepticism towards investigators’ motives and whether the interventions will truly benefit their communities [20, 49, 50, 53]. In the DISCOVER trial, which demonstrated the non-inferior efficacy of emtricitabine/tenofovir alafenamide to emtricitabine/tenofovir disoproxil fumarate, the enrollment of underrepresented and disproportionately affected populations was suboptimal [48, 54]. For example, the percentage of US participants in the study who identified as Black was only 13%, while transgender women comprised less than 1% of the total study sample [48]. The disappointing number of individuals from demographically diverse backgrounds included in DISCOVER and in HIV research overall [47, 48, 55] underscore the need for a multifactorial approach to support actualized engagement with key communities.

However, focusing solely on racism, discrimination, and mistrust oversimplifies the inequities and challenges faced by racial, ethnic, gender, and sexual minorities [32]. Persons from these groups often face competing priorities, including safety, housing, employment, transportation, and mental health [53]. TGNB individuals may face these challenges in addition to others, such as access to gender-affirming care [56]. Advocates have underscored the incorporation of a social justice framework in the design and conduct of research where we acknowledge and address the intersectional oppression that drives disparities and underrepresentation [32, 57]. This motivated our team to rework our approach, collaborate with various stakeholders, and identify strategies to support better representation of communities with a disproportionate HIV burden. Numerous other frameworks that focus on different aspects of community engagement exist, including frameworks for addressing power dynamics between researchers and community members and methods for involving community in establishing research priorities [58]. These strategies to support meaningful stakeholder engagement can be leveraged and adapted to better address the needs and context of future HIV trials and support effective collaboration with community.

The study team followed the principles of GPP [23] and incorporated evidence-informed strategies identified in the literature. In PURPOSE 2, we worked closely with community and patient advocates in the GCAG, as well as with broader stakeholders, to ensure that the study design and procedures were culturally-relevant, inclusive, and responsive to the needs of the populations we wanted to engage. We set enrollment goals that are monitored weekly and shared with site investigators so that recruitment strategies and the order of site activation can be modified as needed. The study team meets internally weekly, with the site investigators and staff monthly, and with the GCAG approximately every two months to discuss study progress, including progress towards the overall demographic goals.

Beyond diversity benchmarks, we aimed to address structural and social factors by selecting research sites in high HIV incidence regions. We partnered with clinics embedded within these areas to increase access to the clinical trial and reduce the potential burdens of participation, such as transportation costs. We were also intentional in selecting sites that had established trust within their communities and with staff demographics reflective of participants. Studies examining participation of underrepresented groups in HIV studies found that individuals were more likely to feel comfortable and engage when research staff were from the same communities [32, 50]. Strategies to increase representation of TGNB individuals and equity in HIV prevention research highlighted the need to evaluate outcomes important to the TGNB community, such as drug-drug interactions between LEN and GAHT, which have been added to the study so the data will be available to guide providers and potential users of PrEP.

Our efforts do have limitations. We acknowledge that requiring diversity trainings for investigators, vendors, internal and external research staff does not always translate to cultural sensitivity and competence. Relatedly, being an investigator or research staff member who shares characteristics with a priority population does not automatically equate to site inclusivity. Most importantly, as enrollment is currently ongoing, we are not yet able to characterize the results of our efforts; but we plan to share the full demographic characteristics of the enrolled PURPOSE 2 trial and whether we achieved our demographic recruitment goals in the future.

## Conclusion

Insights from implementation studies that examined barriers to PrEP uptake have underscored the importance of building trust with communities disproportionately affected by HIV [17]. Underrepresentation of racial, ethnic, and gender minorities in PrEP research have limited our ability to examine outcomes important to these communities and understand potential issues that may be unique to certain populations. Further, inadequate engagement with global stakeholders and community members that have been disproportionately impacted by HIV have hampered PrEP scale-up in key populations and limited its public health impact. Learning from past lessons, we have carefully chosen with whom, where, and how we work to increase diversity, equity, and inclusion in the PURPOSE 2 trial. While these are initial steps and further work is needed to achieve equitable representation, our efforts to increase engagement of Black and Hispanic/Latinx MSM and TGNB individuals in the PURPOSE 2 study may serve as framework for future PrEP and other clinical trials.
